# Comparison of the Effects of Different Cryoprotectants on Stem Cells from Umbilical Cord Blood

**DOI:** 10.1155/2016/1396783

**Published:** 2015-12-07

**Authors:** Gecai Chen, Aihuan Yue, Zhongbao Ruan, Yigang Yin, Ruzhu Wang, Yin Ren, Li Zhu

**Affiliations:** ^1^Department of Cardiology, Taizhou People's Hospital, 399 Hailing Road, Taizhou, Jiangsu 225300, China; ^2^Stem Cell Research Center, 399 Hailing Road, Taizhou, Jiangsu 225300, China

## Abstract

*Purpose*. Cryoprotectants (CPA) for stem cells from umbilical cord blood (UCB) have been widely developed based on empirical evidence, but there is no consensus on a standard protocol of preservation of the UCB cells. *Methods*. In this study, UCB from 115 donors was collected. Each unit of UCB was divided into four equal parts and frozen in different kinds of cryoprotectant as follows: group A, 10% ethylene glycol and 2.0% dimethyl sulfoxide (DMSO) (v/v); group B, 10% DMSO and 2.0% dextran-40; group C, 2.5% DMSO (v/v) + 30 mmol/L trehalose; and group D, without CPA. *Results*. CD34^+^, cell viability, colony forming units (CFUs), and cell apoptosis of pre- and postcryopreservation using three cryoprotectants were analyzed. After thawing, significant differences in CD34^+^ count, CFUs, cell apoptosis, and cell viability were observed among the four groups (*P* < 0.05).  *Conclusion*. The low concentration of DMSO with the addition of trehalose might improve the cryopreservation outcome.

## 1. Introduction

In 1988, umbilical cord blood (UCB) was first successfully used as a source of stem cells for hematopoietic reconstitution in a 5-year-old boy with Fanconi anemia, an inherited bone-marrow-failure syndrome that could be cured only by allogeneic hematopoietic stem cell transplantation (HSCT) in Paris, France, by Dr. Eliane Gluckman and her colleagues. In recent decades, UCB stem cells have been used for the treatment of malignant diseases, such as hematological malignancies [1–3], Hurler syndrome [4, 5], Krabbe disease [6], primary immunodeficiency diseases [7], bone marrow failure [8], and beta thalassemia [9]. With in-depth research on UCB, more and more patients have been able to benefit from UCB stem cells, and HSCT is now performed all over the world. In 2009, the U.S. Food and Drug Administration (FDA) published “Guidance for Industry: Minimally Manipulated, Unrelated Allogeneic Placental/Umbilical Cord Blood Intended for Hematopoietic Reconstitution for Specified Indications” [10]. Many public or private UCB banks have now been established around the world for the collection and cryopreservation of UCB units.

UCB is cryopreserved in liquid nitrogen and is recovered when needed. During cryopreservation and thawing, the main cause of cell death is not long-term storage at low temperatures, but the processes of both cooling and warming through a range of temperatures, such as from −15 to −60°C. Ice crystal formation can also be reduced by the addition of sulfoxides or alcohols such as DMSO. DMSO freely permeates cell membranes due to its low hydrophilicity and molecular weight and is therefore thought to disrupt ice crystal nucleation and formation by forming hydrogen bonds with water [11]. To minimize cellular damage, different concentrations of DMSO combined with polysaccharides are used for UCB cryopreservation. Nicoud et al. utilized an intracellular-like media with DMSO (≤5%) in the frozen cord blood and gained equivalent or slightly better postthaw recoveries than cryopreservation solution with DMSO (10%) [12]. There seems to be little adverse effect on cell recovery or engraftment in reducing DMSO concentration to 5% at optimal cooling rates [13], and concentrations as low as 2% combining disaccharides or polysaccharides have been successfully employed [14].

Due to its stability upon freezing, disaccharides such as trehalose have been investigated as a CPA [15]. Alternative cryoprotectants such as hydroxyethyl starch and trehalose, either in combination with DMSO or alone, have also been shown to be effective in cryopreserving haematopoietic cells [16]. Various concentrations of DMSO or trehalose with or without addition of insulin were compared. Trehalose exerts a similar cryoprotective potential for hematopoietic progenitor and stem cells like large impermeant sugars and could possibly replace DMSO at least in part as cryoprotectant in the setting of hematopoietic cell transplantation [15]. Trehalose was considered nontoxic cryoprotective agents on the viability of cord blood-derived mononuclear cells [17]. Wang et al. used DMSO-free CPA solutions which contained ethylene glycol (EG), 1,2-propylene glycol (PG), and sucrose as basic CPAs and results showed that the viability of thawed umbilical cord blood-derived mesenchymal stem cells was enhanced [18]. Dextran and DMSO were used as cryoprotectants in many cord blood banks [19].

Assessment of stem cell content and viability after long-term storage is a critical step before successful stem cell transplantation. In this study, the three cryoprotectants were assessed. Most cord blood banks currently use 10% ethylene glycol (EG) and 2.0% DMSO (v/v) [20], 10% DMSO (v/v) and 2.0% dextran-40 [21], and 2.5% DMSO (v/v) + 30 mmol/L trehalose [21]. 115 UCB units were processed and cryopreserved. Each unit was divided as follows: A, 10% ethylene glycol (EG) and 2.0% DMSO (v/v) [20]; B, 10% DMSO (v/v) and 2.0% dextran-40; C, 2.5% DMSO (v/v) + 30 mmol/L trehalose; and D, without CPA. 103 qualified UCB units were analyzed at each of the data points. Cell apoptosis, a colony forming unit (CFU) assay, and CD34^+^ cell count and cell viability were analyzed both before and after cryopreservation. The data showed that group C exhibited higher cell viability and CFUs and a lower apoptosis rate after thawing than either group A, B, or D.

## 2. Materials and Methods

### 2.1. Processing UCB

UCB was obtained from healthy, full-term, naturally delivered newborns. Written informed consent was obtained from the mothers and their family members. The protocols were reviewed and approved by the Review Board and Ethics Committee of People's Hospital of Taizhou, Jiangsu, China. UCB units were processed within 4.0 h of collection. UCB weight was determined by weighing the collection bag and contents and then subtracting the weight of the empty collection bag and the citrate-phosphate-dextrose-adenine (CPDA) anticoagulant solution. A 10 mL UCB sample was gently mixed with 10 mL saline. Ten-milliliter lymphocyte separation medium (LSM1.077) was poured into a 50 mL tube. The 20 mL cell suspension on top of the separation medium was carefully added to the tube without disturbing the interphase. The tube was centrifuged at 440 ×g for 40 min. Most of the supernatant was then aspirated without disturbing the layer of mononuclear cells in the interphase. The mononuclear cells were then aspirated from the interphase, washed with saline, and centrifuged at 360 ×g for 10 min. The excess red blood cells and plasma were removed.

### 2.2. Sterility Testing of UCB (Precryopreservation)

The plasma was detected using the BD BACTEC 9120 (BD Biosciences) Blood Culture System. The outer surface of the Standard/10 Aerobic/F and Lytic/10 Anaerobic/F (BD Biosciences, Sparks, MD, USA) was cleaned with 75% alcohol. The plastic flip cap was removed and the exposed rubber septum was cleaned with an alcohol swab. Then, 3.0–10 mL plasma from UCB was collected for anaerobic bacteria and fungi cultures and another 3.0–10 mL for aerobic bacteria and fungi cultures. The inoculated culture vials were loaded into the instrument, and a temperature of (35 ± 1.5)°C in the racks and (30 ± 1.0)°C within the cabinet was maintained. The plasma was cultured for 7 d. The control assays were carried out by sterile saline for negative control and* Escherichia coli* ATCC25922 for positive control.

### 2.3. UCB Cryopreservation and Thawing

Each UCB unit was divided into four parts and each part was cryopreserved in one of the three cryoprotectants described above, which were added before the UCB units were frozen. A controlled-rate freezer (CRF) was used to slowly freeze the prepared stem cells to a temperature of −80°C. The following freezing protocol was used for cryopreservation of the stem cells subsequent to processing: wait at 4.0°C, stage 1: ramp 1.0°C/min until sample = −5.0°C; stage 2: ramp 21°C/min until chamber = −54.0°C; stage 3: ramp 17°C/min until chamber = −21.0°C; stage 4: ramp 2.0°C/min until sample = −40.0°C; and stage 5: ramp 10°C/min until sample = −80.0°C. After freezing, the units were immediately transferred from CRF to a liquid nitrogen vessel for storage.

Six months after cryopreservation, the UCB units were retrieved from the liquid nitrogen and placed into a water bath at 37°C. To accelerate thawing, the units were carefully moved through the water and their contents were gently kneaded. As soon as the contents had thawed, the sample was removed from the water bath. Five minutes were allowed for equilibration. The tube was then centrifuged at 3000 revolutions per minute (rpm) for 5 min. After centrifugation, the supernatant was discarded by pipettor gently, except group D. A five classification hematology analyzer (Beckman Coulter, Inc., Brea, CA, USA) was used for counting the total nucleated cells (TNCs) and the recovery of TNCs was calculated. Before freezing, the UCB unit was to have > 5.0 × 10^8^ TNCs. TNC = white blood cell (WBC) + nucleated red blood cell (nRBC). The control assays were carried out for WBC (Coulter 5C Cell Control, 7547001, Beckman Coulter) and nRBC (LH-nRBC, LH004, R&D). 103 qualified UCB units were analyzed at each of the data points.

### 2.4. CD34^+^ Count and Cell Viability of Hematopoietic Stem Cells (Pre- and Postcryopreservation)

10 *μ*L CD45-FITC Ab, 10 *μ*L CD34-PE Ab reagent, and 10 *μ*L7-AAD reagent (BD Biosciences, Sparks, MD, USA) were pipetted into a tube, and a 50 *μ*L well-mixed sample was pipetted to the bottom of the tube. The tube was placed in a vortex, protected from light, and incubated at room temperature for 15 min. After incubation, 1.0 mL 1x lysing reagent was added to the tube. The tube was vortexed again and incubated at room temperature for 10 min. After incubation, the tubes were centrifuged at 300 ×g for 5.0 min. The supernatant was discarded and 1.0 mL PBS was added to each tube, the contents were mixed, and the tubes were centrifuged at 300 ×g for 5 min. The supernatant was again discarded, 350 *μ*L PBS was added to each tube, and the contents were mixed. The UCB units were tested using BD FACSCanto II (BD Biosciences, Sparks, MD, USA). The control assay was carried out for CD34^+^ count (BD Stem Cell Control Kit, 340991, BD). The UCB units in which the CD34^+^ cells were >0.25% TNCs and the viability of TNCs was >85% before freezing were chosen. 103 qualified UCB units were analyzed at each of the data points.

### 2.5. Colony Forming Units (Pre- and Postcryopreservation)

One-tenth milliliter UCB was placed in a sterile EP tube, 1.0 mL NH_4_Cl was added to the sample, and the solution was mixed and left to rest at room temperature for 10 min. The sample was then centrifuged at 300 ×g for 5 min. The supernatant was removed, 1.0 mL DMEM culture medium was added, and the contents were mixed and washed. The supernatant was again removed and DMEM was added to the suspended cells. To perform a cell count, 0.1 mL cell suspension was removed. The stem cells were cultured in 1.2 mL MethoCult GF H4434 (STEMCELL Technologies, Canada) culture medium. The final concentration was 1 × 10^5^ cells/mL. Two wells of each sample were seeded in a 24-well plate with 0.5 mL cell suspension. The 24-well cell culture was placed in a humidified atmosphere with 5.0% CO_2_ at 37°C. After culturing for 14–16 d, the culture board was removed and an inverted microscope was used to count the colonies. The following standards were followed to count colonies: granulocytic, monocytic (GM) ≥ 30 cells/colony; granulocyte, erythrocyte, monocyte, and megakaryocyte (GEMM) ≥ 40 cells/colony; and burst-forming unit-erythroid (BFU-E) ≥ 50 cells/colony. 103 qualified UCB units were analyzed at each of the data points.

### 2.6. Cell Apoptosis Analysis Using Annexin V and Propidium Iodide Staining (Pre- and Postcryopreservation)

0.1 mL sample of UCB was placed into an EP tube and 1.0 mL NH_4_Cl was added to the sample. The sample was washed twice with PBS, and the cell concentration was adjusted to 2.0 × 10^6^ cells/mL. 1.0 × 10^6^ cells/mL were then centrifuged for 5.0 min at 300 ×g, the supernatant was discarded, and the cells were stained with 1.0 *μ*g/mL annexin V and propidium iodide (PI). The cells were then incubated for 30 min at 4.0°C and fluorescence was measured by flow cytometry. 103 qualified UCB units were analyzed at each of the data points.

### 2.7. Statistical Analyses

Data were expressed as the mean ± SEM. Groups A, B, C, and D were compared using the analysis of variance (ANOVA). A 5.0% probability (*P* < 0.05) was used as the level of statistical difference.

## 3. Results

### 3.1. Recovery of Viable TNC (after Thawing)

**Figure 1:**
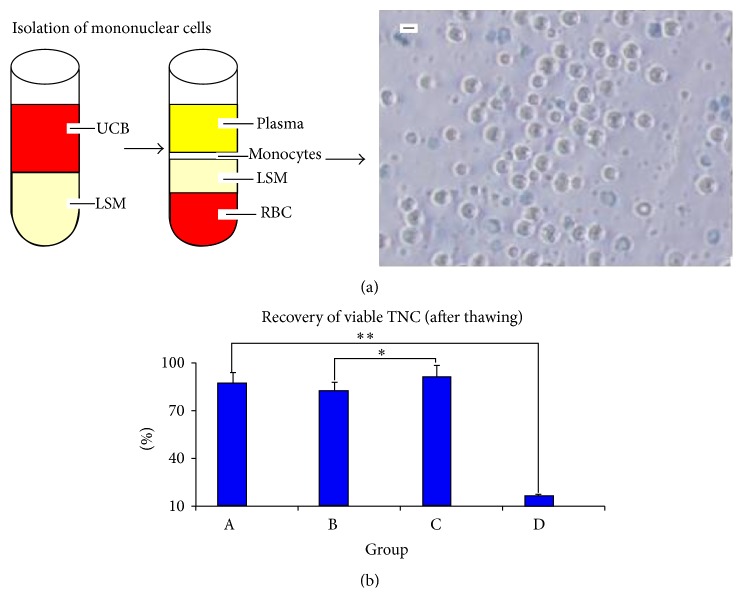
(a) The mononuclear cells were separated from UCB by density gradient centrifugation. (b) Recovery of viable TNC (after thawing): the viable TNC recovery of group C was higher than that of either groups A, B (*P* < 0.05) or group D (*P* < 0.01). The recovery of group D was lower than that of either group A, B, or C (*P* < 0.01). Scale bars: 5 *μ*m.

The gross weight of the UCB collection bags minus the weight of the collection bag itself and CPDA was the gross weight of UCB. UCB units > 100 mL were chosen for study. The average volume of UCB was (122.8 ± 17.8) mL. The mean TNC was (11.3 ± 3.4) × 10^8^ after processing. The recovery of viable TNC in the four different groups (groups A, B, C, and D) was (87.35 ± 6.52)%, (82.43 ± 5.51)%, (91.18 ± 7.40)%, and (16.15 + 1.42)% after thawing, respectively. The viable TNC recovery of group C was higher than that of either group B (*P* < 0.05) or group D (*P* < 0.01). The recovery of group D was lower than that of either group A, B, or C (*P* < 0.01) (Figure 1).

### 3.2. UCB Sterility (Precryopreservation)

Five UCB units were contaminated with anaerobic bacteria. The BD BACTEC9120 system showed a typical S-shaped growth curve (see Supplementary Information in Supplementary Material available online at http://dx.doi.org/10.1155/2016/1396783). Because of the contaminated samples, the CFU assay could not be completed for these units and the positive samples were discarded.

### 3.3. CD34^+^ Count and Cell Viability of TNCs (Pre- and Postcryopreservation)

**Figure 2:**
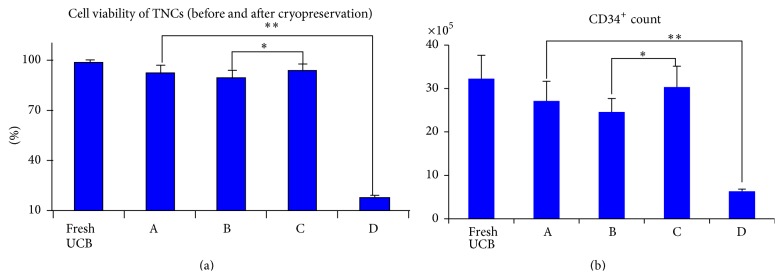
(a) The cell viability of group C was higher than that of groups A, B (*P* < 0.05) and group D (*P* < 0.01) after thawing. (b) The CD34^+^ count of group C was higher than that of groups A, B (*P* < 0.05) and group D (*P* < 0.01) after thawing.

The mean count of CD34^+^ was (32.25 ± 5.37) × 10^5^ and cell viability was (98.34 ± 1.23)% (fresh UCB). After thawing, the mean count of CD34^+^ was (27.13 ± 4.51) × 10^5^ (group A), (24.57 ± 5.12) × 10^5^ (group B), (30.34 ± 4.78) × 10^5^ (group C), and (6.3 ± 0.51) × 10^5^ (group D). A visible difference in the CD34^+^ count among the four groups (*P* < 0.05) was noted, with group C being the highest. The mean percentages of cell viability after thawing were (92.35 ± 5.26)% (group A), (89.43 ± 5.12)% (group B), (94.18 ± 3.97)% (group C), and (18.13 ± 0.98)% (group D). The cell viability of group C was higher than that of either groups A, B (*P* < 0.05) or group D (*P* < 0.01) (Figure 2).

### 3.4. CFU (Pre- and Postcryopreservation)

**Figure 3:**
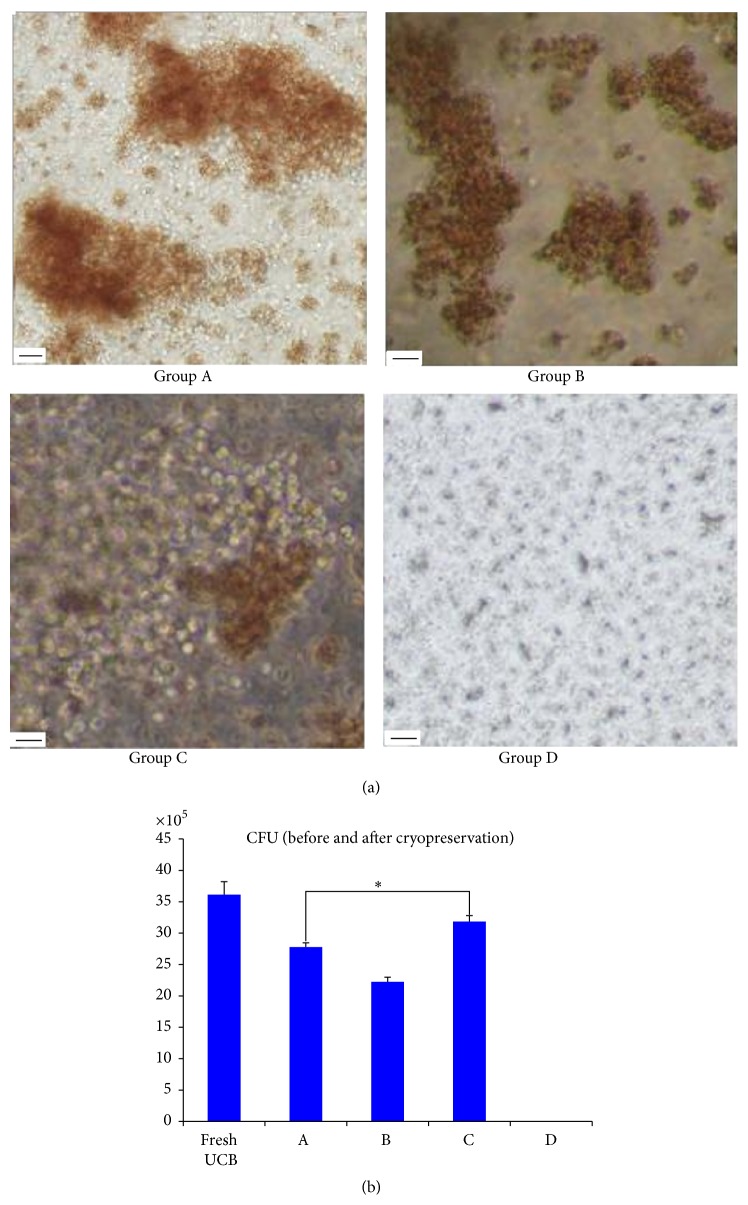
(a) The colonies were observed in an inverted microscope. CFU-GEMM is full of the immature nucleated red blood cells and granulocytes from the center to the edge of clone and two CFUs of GEMM were showed in group A. BFU-E are red and were shown in group B. CFU-GM is almost transparent and BFU-E and CFU-GM were shown in group C. There was almost no colony formation in group D. (b) The CFU of group C was higher than that of group A (*P* < 0.05) and groups B, D (*P* < 0.01) after thawing. Scale bars: 100 *μ*m.

When TNCs were plated at 1.0 × 10^5^ cells/mL, the average CFU was (36.14 ± 2.06) × 10^5^ (fresh UCB). After thawing, the average CFU in the five different cryoprotectants was (27.78 ± 0.58) × 10^5^ (group A), (22.25 ± 0.52) × 10^5^ (group B), (31.86 ± 0.64) × 10^5^ (group C), and (0.00 ± 0.00) × 10^5^ (group D). There was almost no colony formation in group D. The CFU of group C was higher than that of either group A (*P* < 0.05) or groups B, D (*P* < 0.01) (Figure 3).

### 3.5. Cell Apoptosis Analysis (Pre- and Postcryopreservation)

**Figure 4:**
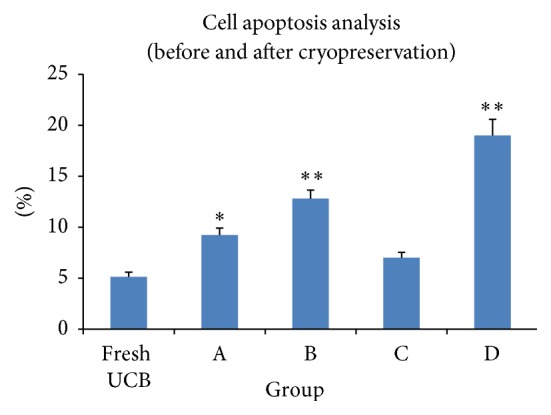
The cell apoptosis of group C was lower than that of group A (*P* < 0.05) or groups B, D (*P* < 0.01) after thawing.

Apoptosis of TNCs before cryopreservation and thawing after cryopreservation with one of the three cryoprotectants were analyzed using annexin V/PI staining. The mean percentages of cell apoptosis were (5.13 ± 0.45)% (fresh UCB), (9.24 ± 0.68)% (group A), (12.82 ± 0.83)% (group B), (7.01 ± 0.52)% (group C), and (19.01 ± 2.57)% (group D). The cell apoptosis of group C was lower than that of either group A (*P* < 0.05) or groups B, D (*P* < 0.01) (Figure 4).

## 4. Discussion

Current UCB cryoprotectants have been developed from empirical evidence; however, there is no consensus on a standard protocol of preservation. Different UCB banks have adopted the use of different cryoprotectants. In recent years, the cryoprotectants were 10% EG and 2.0% DMSO (v/v) [20], 10% DMSO and 2.0% dextran-40 [21], and 2.5% DMSO (v/v) + 30 mmol/L trehalose [21], which were used in this study for groups A, B, and C, respectively. Regardless of which cryoprotectant was used, the goal was to minimize cell damage or cell death. Because both slow and rapid cooling are detrimental to cells, cryopreservation protocols for UCB stem cells should adopt an optimal cooling rate and a cryoprotectant, which would reduce ice formation until the intracellular water achieves a “glassy state” [22]. Cryoprotectants, such as glycerol, DMSO, and EG, are excellent; they have low molecular weights and effectively penetrate into the cells and prevent intracellular ice formation. Trehalose lacks natural permeability to human cell membranes and the development of novel methods for efficient intracellular delivery of trehalose has been an ongoing investigation [23]. There is a consensus on the fact that, during warming, a rapid warming rate is required to prevent ice recrystallization and quickly remove the cryoprotectant to minimize toxicity to the cell [24].

In this study, UCB was collected from 115 donors; five were discarded because of bacterial contamination and seven were discarded because of low CD34^+^ counts. The 103 qualified UCB units were divided into four groups and cryopreserved with one of the three different cryoprotectants. In CD34^+^ testing by FCM, the antibody could bind antigen, whether cells are dead or alive. But dead cells are more easily broken in potent hemolysin (containing sodium azide), so CD34^+^ cell count of group D was lower than that of group C (*P* < 0.01). Combined with cell viability and apoptosis assay, although some of the cells were alive, which had started apoptosis, CD34^+^ cells, cell viability, and CFUs were significantly higher in group C than in either of the other two groups. The results demonstrated that BFU-E were most likely to be affected during cryopreservation and recovery process. The outcomes were consistent with those reported by Motta et al. [25–27].

Several variables including the CPA concentration, composition, cooling rate, thawing rate, and hold temperatures all matter regarding the quality of UCB. The cryoprotectant in popular use is DMSO, and the use of a controlled rate freezing technique at 1 to 2°C/min and rapid thawing is considered standard. The standard temperatures currently in use are −196 to −80°C and the currently recommended optimal storage conditions are in the vapor nitrogen phase, at −156°C [14]. −156°C was used in this study. In controlled rate freezing, the concentrated stem cells are frozen down at a rate of 1-2°C/min up to a temperature point of about −40°C [28]. Our freezing protocol was as follows: stage 1: ramp 1.0°C/min until sample = −5.0°C; stage 2: ramp 21°C/min until chamber = −54.0°C; stage 3: ramp 17°C/min until chamber = −21.0°C; stage 4: ramp 2.0°C/min until sample = −40.0°C; and stage 5: ramp 10°C/min until sample = −80.0°C.

Trehalose has also been adopted as a cryoprotective agent for mesenchymal stromal cells and porcine spermatogonial stem cells [29, 30]. In addition to cell toxicity, DMSO inactivates cisplatin, carboplatin, and other platinum complexes [31]. Moreover, cryopreservation with DMSO prevents accurate analysis of mitochondrial respiration in skinned skeletal muscle fiber [32].

## 5. Conclusions

The use of trehalose was shown to result in improved cell survival and differentiation capacity after thawing. A better understanding of the behavior and properties of UCB provides a valuable resource for understanding cryoprotective agents during cryopreservation. After thawing, group C, containing 2.5% DMSO (v/v) + 30 mmol/L trehalose, was shown to improve the survival of UCB stem cells compared to the other two groups prepared without trehalose.

## Supplementary Material

Legend: Five UCB units were contaminated by anaerobic bacteria. a) One of typical S-shaped growth curve was showed. b) The staphylococcus was discriminated by Gram staining in corresponding UCB
