# Supplementary figures and images for: Comparison of the Effects of Different Cryoprotectants on Stem Cells from Umbilical Cord Blood
Source: Stem Cells Int. 2015 Dec 7;2016:1396783. doi: 10.1155/2016/1396783 (PMC4685149; doi:10.1155/2016/1396783)

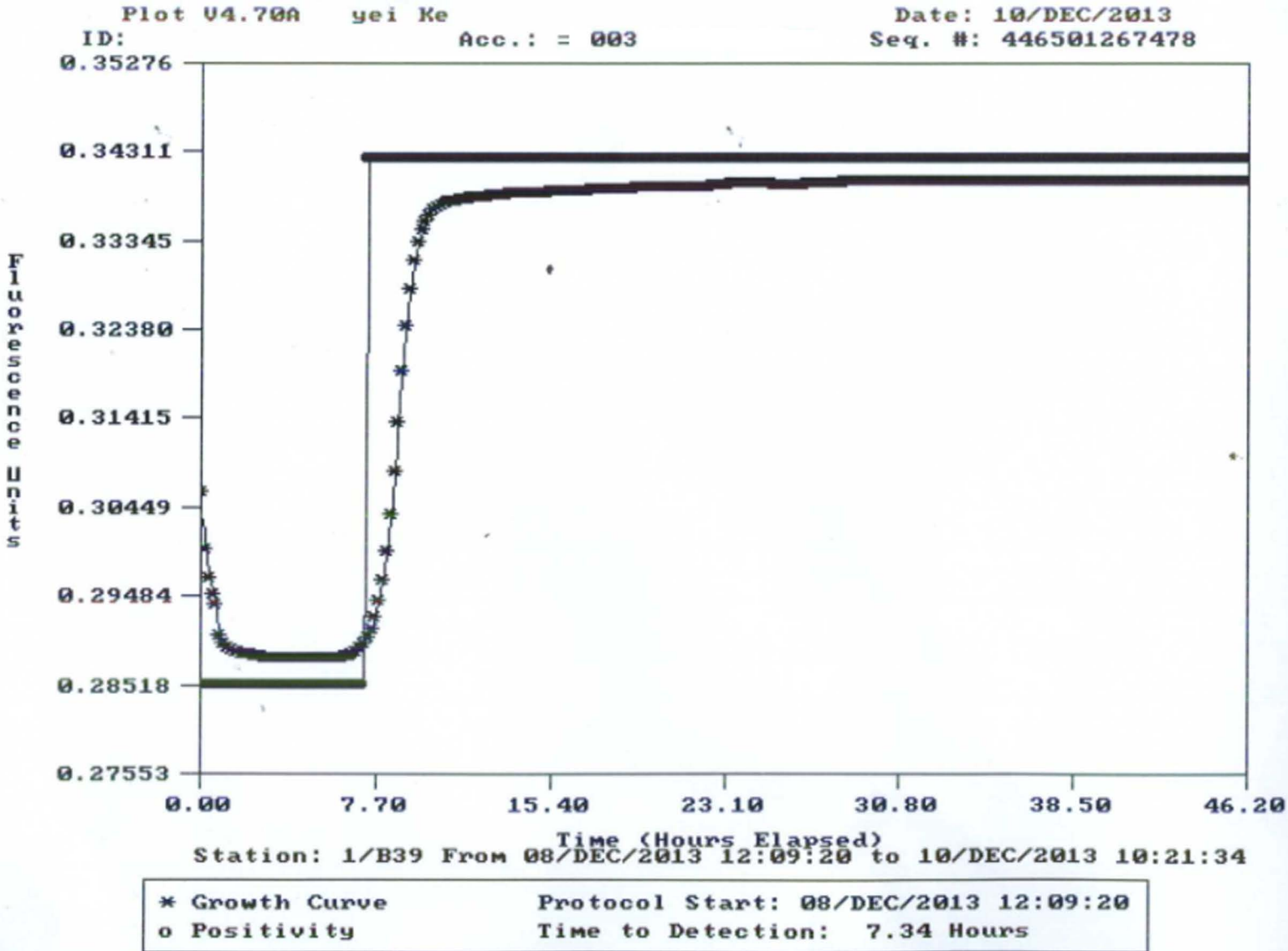

a

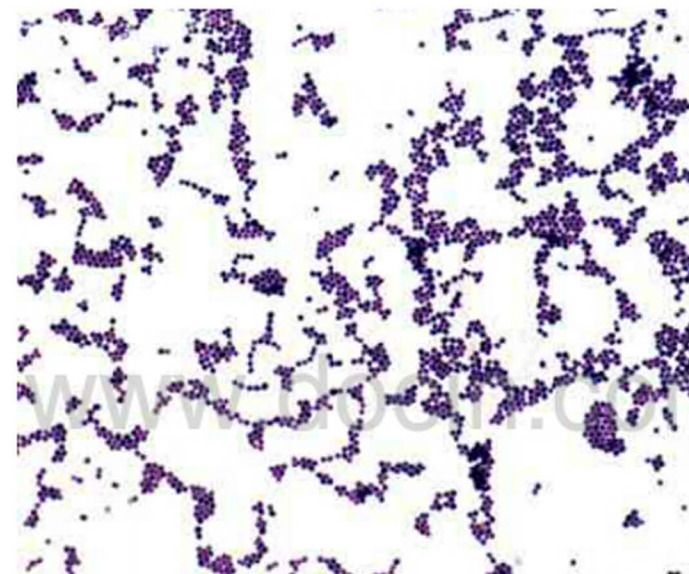

10 × 100

b

Supplement: Supplementary file 1 — Legend: Five UCB units were contaminated by anaerobic bacteria. a) One of typical S-shaped growth curve was showed. b) The staphylococcus was discriminated by Gram staining in corresponding UCB [file 1396783.f1.pdf]
